# Supplementary material for: The potential impact of declining development assistance for health on population health in Malawi: A modelling study
Source: PLoS Med. 2025 Aug 21;22(8):e1004488. doi: 10.1371/journal.pmed.1004488 (PMC12370021; doi:10.1371/journal.pmed.1004488)
Supplement: S1 Text — (DOCX) [file pmed.1004488.s001.docx]

**Modelling HRH constraints**

The TLO model self-consistently captures the number of specific appointment types (referred to as “health-system interactions” or HSIs) requested on any given day at every modelled facility. To enforce HRH constraints on the delivery of these requested health services, the model relies on two key assumptions: A) the standard patient-facing time required from each medical cadre to deliver each type of HSI, and B) The total patient-facing time available from each cadre at each facility on any given day. Based on these assumptions, the model can ensure that HSIs are only delivered until exhaustion of available capabilities, as discussed in detail in [[1].](#_bookmark41) In the model, the former

(A) is informed by consensus estimates on the time needed for the delivery of each service as reported by the Human Resources for Health Strategic Plan (HRH SP) 2018–2022 [[2],](#_bookmark50) while the latter (B) is informed by the Detailed Annex for the Health Workforce Interventions of the Malawi Health Sector Strategic Plan (HSSP III) for 2023-2030 (HSSP III HRH Annex) [[3,](#_bookmark51) [4].](#_bookmark32)

While these sources provide the most reliable theoretical expectation of both A) and B) in the country, a number of factors may alter them in practice (see discussion in [[1]).](#_bookmark41) For example, the level of experience of HCWs may be reasonably expected to impact the duration of appointments, while HCWs may cope with a large volume of requests for treatment by shortening the expected appointment duration and/or working overtime. Combined, these and other factors may result in a “productivity level” which differs from the one expected from assumptions around A) and B) alone.

In order to capture these effects while still enforcing HRH constraints in this analysis, we adopt the following approach. The *Thanzi La Onse* model is calibrated to epidemiological and health system data in the period 2015- 2019, and reproduces the observed number of HSIs delivered by the healthcare system during this period [[5].](#_bookmark25) By multiplying the number of HSIs delivered with the assumed time requirement from each medical cadre for each type of HSI (averaged over a year), we can estimate the average number of minutes of patient-facing time *effectively* delivered by each HCW type on any given day of that year. This represents the “effective” HRH capabilities available in the country, which indeed surpass those estimated by the HSSP III HRH Annex [[4].](#_bookmark32) Finally, we replace assumptions around B) with the so-estimated effective capabilities in 2018, the year before capabilities expansion is considered, and in doing so account for the real-world productivity of health workers in service delivery (as opposed to theoretical productivity levels expected), while enforcing HRH constraints in subsequent years.

**References**

1. Molaro M, Mohan S, She B, Chalkley M, Colbourn T, Collins JH, et al. A new approach to Health Benefits Package design: an application of the Thanzi La Onse model in Malawi. PLOS Computational Biology. 2024;20(9):e1012462. Available from: [https://doi.org/10.](https://doi.org/10.1371/journal.pcbi.1012462) [1371/journal.pcbi.1012462](https://doi.org/10.1371/journal.pcbi.1012462).
2. Berman L, Prust ML, Maungena Mononga A, Boko P, Magombo M, Teshome M, et al. Using modeling and scenario analysis to support evidence-based health workforce strategic planning in Malawi. Hum Resour Health. 2022 Apr;20(1):34.
3. Government of Malawi. Detailed Annex for the Health Workforce Interventions of the Malawi Health Sector Strategic Plan (HSSP III) for 2023-2030. Government of Malawi: Ministry of Health; 2023. Available from: [https://www.health.gov.mw/wp-content/uploads/](https://www.health.gov.mw/wp-content/uploads/2023/06/1L-HSSP-III-HRH-Annex.pdf) [2023/06/1L-HSSP-III-HRH-Annex.pdf](https://www.health.gov.mw/wp-content/uploads/2023/06/1L-HSSP-III-HRH-Annex.pdf).
4. She B, Mangal TD, Prust ML, et al. Health workforce needs in Malawi: analysis of the Thanzi La Onse integrated epidemiological model of care. PREPRINT (Version 1). 2024 July. Available at Research Square. Available from: [https://doi.org/10.21203/rs.](https://doi.org/10.21203/rs.3.rs-4770323/v1) [3.rs-4770323/v1](https://doi.org/10.21203/rs.3.rs-4770323/v1).
5. Hallett TB, Mangal TD, Tamuri AU, Arinaminpathy N, Cambiano V, Chalkley M, et al. Estimates of resource use in the public-sector health- care system and the effect of strengthening health-care services in Malawi during 2015–19: a modelling study (Thanzi La Onse). The Lancet Global Health. 2024 Nov. Available from: <https://www.sciencedirect.com/science/article/pii/S2214109X24004133>
