# Supplementary material for: The potential impact of declining development assistance for health on population health in Malawi: A modelling study
Source: PLoS Med. 2025 Aug 21;22(8):e1004488. doi: 10.1371/journal.pmed.1004488 (PMC12370021; doi:10.1371/journal.pmed.1004488)
Supplement: S2 Text — (DOCX) [file pmed.1004488.s002.docx]

**Effect of consumable availability**

In the analysis presented we relied on an assumption of perfect consumable availability over the entire period considered. In this section, we conduct a sensitivity analysis to establish the impact that deviations from this assumption would have on the results. To do so, we evaluate the same scenarios under an assumption of present-day consumable availability (see [[1]](#_bookmark25) for details).

In Fig B.4 we compare how total DALYs incurred scale with yearly expenditure growth under the two assumptions of consumable availability. The return in health as a function of expenditure, which as expected is systematically lower in the case of present-day consumable availability, is largely unaffected by the assumption of consumable avail- ability under the two assumptions. As a result of the higher health burden incurred under a present-day consumable availability, however, the percentage loss incurred under the scenarios forecasted by the IHME is slightly lower than in the case of perfect consumables (12.7 (95% CI [11.4, 13.9]) and 5.4 (95% [4.4, 6.0])) for the lower and upper bound IHME forecasts respectively).

In Fig [B.5,](#_bookmark16) on the other hand, we consider the impact of consumable availability on the evolution of the health burden as a function of the yearly expenditure growth for important areas of health.


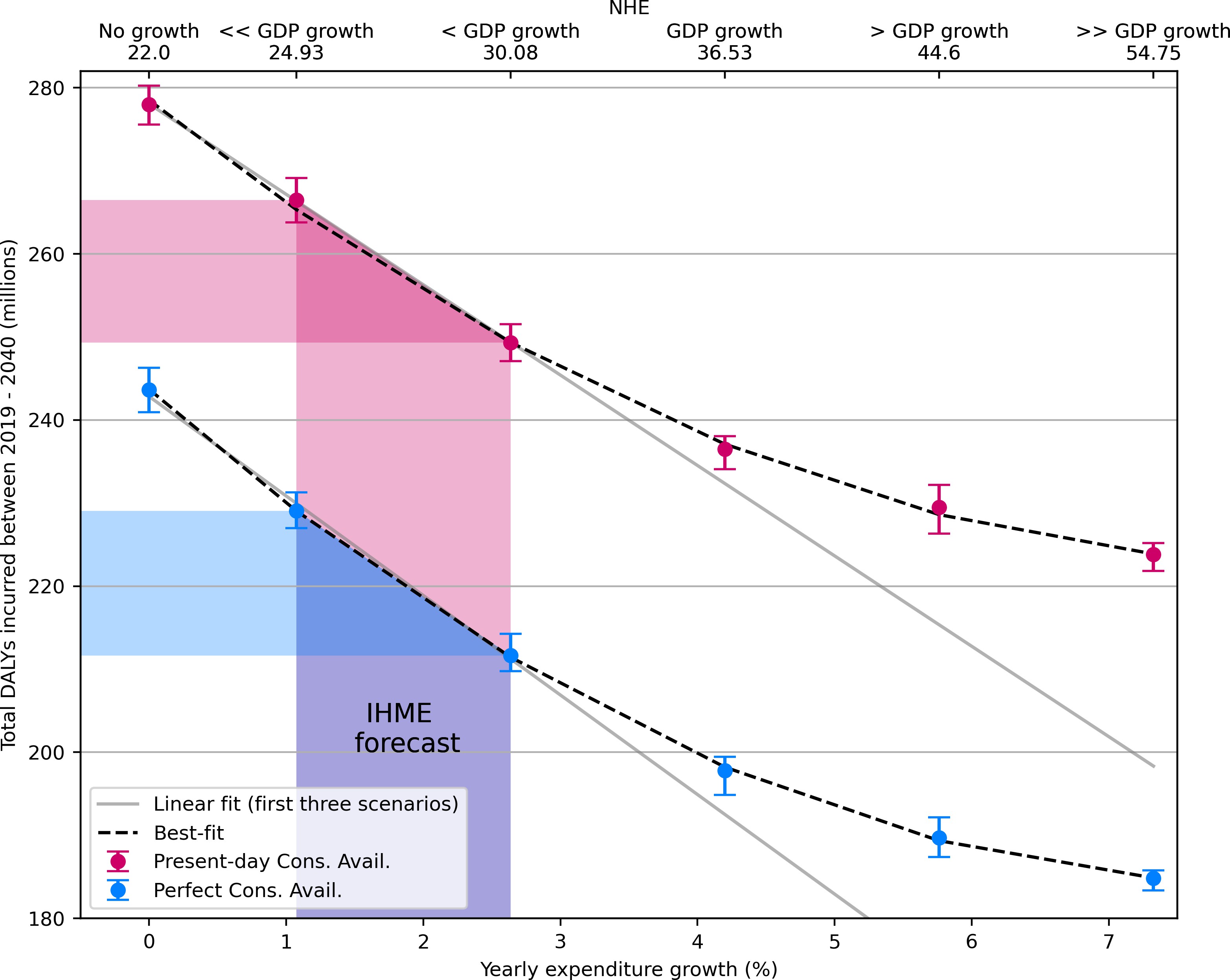

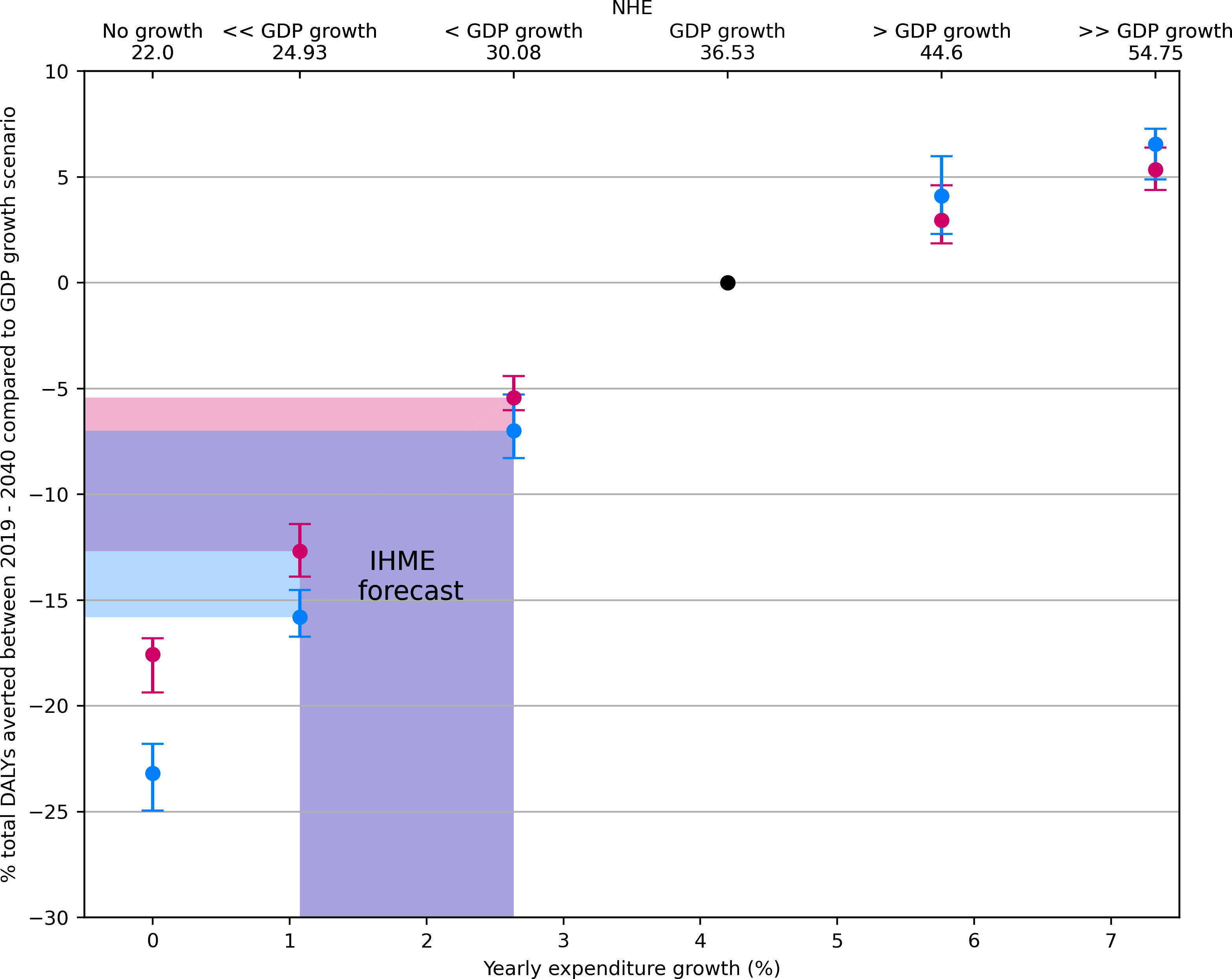


Fig B.4: *Left plot:* Total DALYs incurred in the period 2019–2040 (inclusive) as a function of the yearly expenditure growth, as well as the normalised total expenditure (NHE) over that period under each scenario (top x-axis), as defined in Eqn. [2,](#_bookmark3) for two cases of consumable availability: present-day (magenta points) and perfect (blue points) consumable availability. *Right plot:* Percentage DALYs averted in the same period compared to the consumable-availability specific “GDP growth” scenario. both plots, points represent mean values, while error bars indicate the 95% Cis defined in the Methods section.


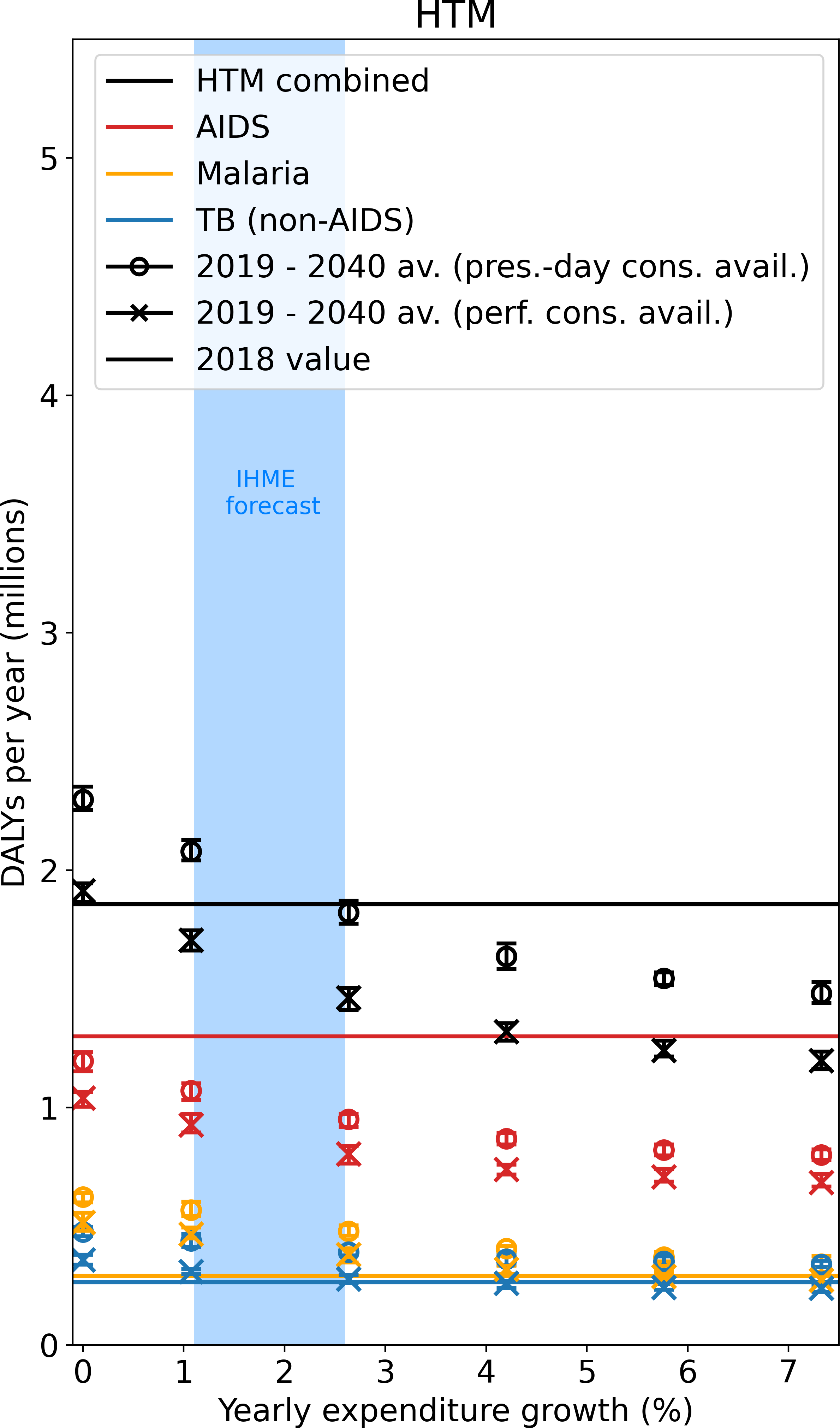

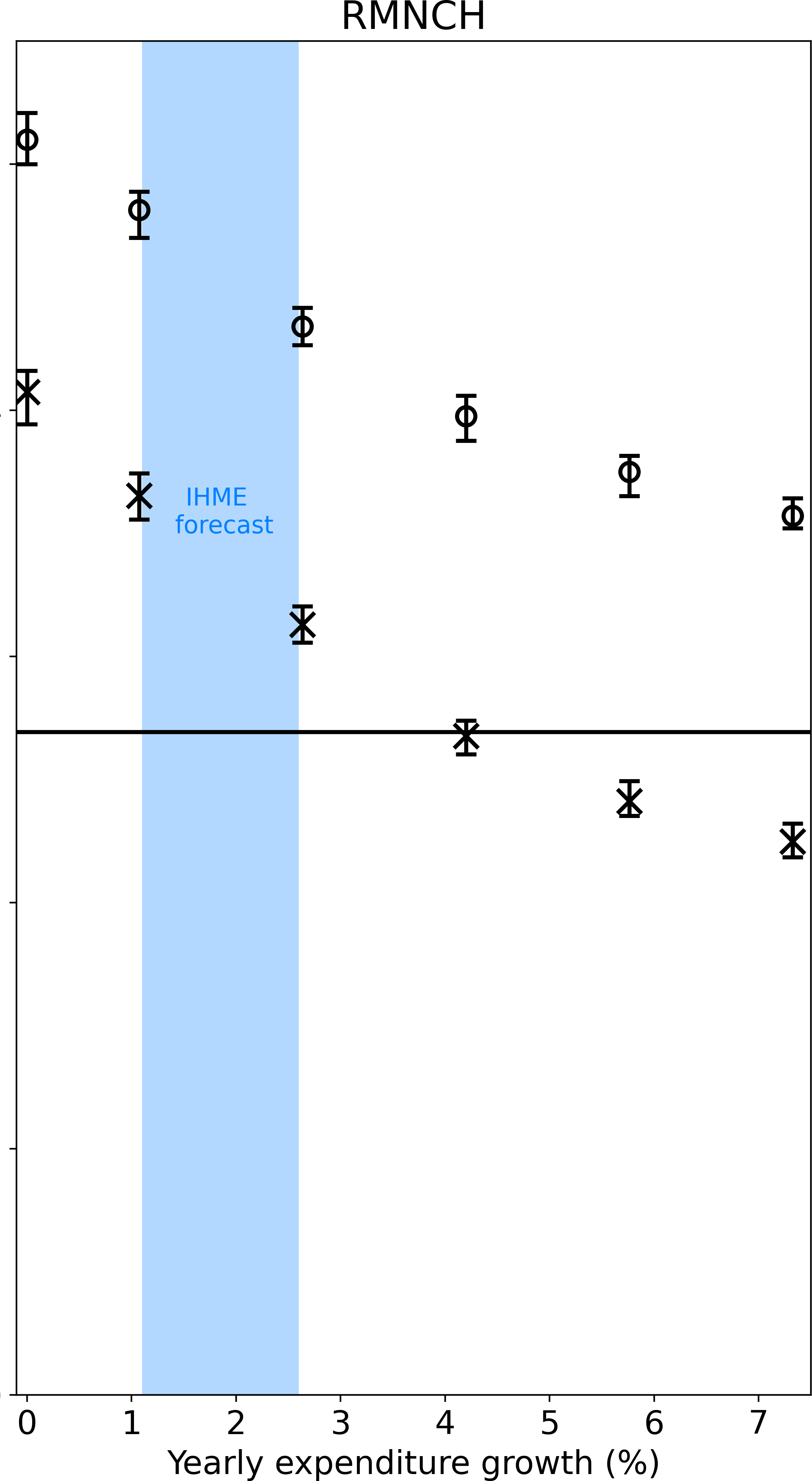

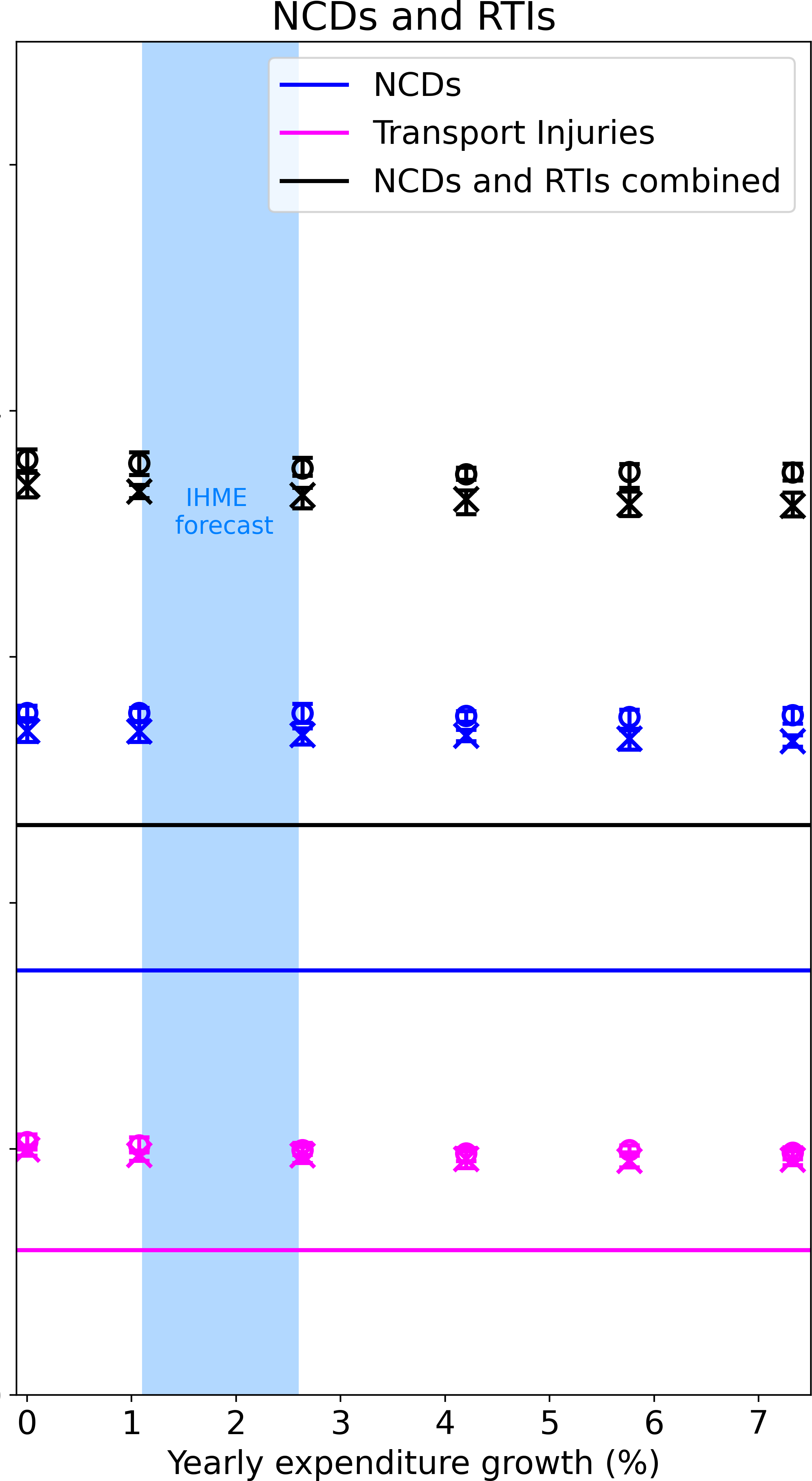


Fig B.5: Average yearly DALYs incurred between 2019-2040 (inclusive), grouped into three meaningful categories (see the Results section), for two cases of assumed consumable availability: the circles show the present-day consumable availability case, while the crosses show the perfect consumable availability case. In all plots, points represent mean values, while error bars indicate the 95% CIs defined in the Methods section. Horizontal lines indicate the yearly DALYs burden for each cause in 2018. This means that any scenario *above* the respective 2018 level incurred, on average, a worsening of the health burden due to that cause over the 2019-2040 period, whereas any scenario *below* the respective 2018 level incurred an improvement.

In the case of HTM, the effect of consumable availability on the health burden is modest. This is due to the already high consumable availability for diseases mainly funded through vertical programmes [[2].](#_bookmark52) It does however suggest that, should present-day consumable availability levels persist throughout the entire period, a minimum of “< GDP growth” expenditure should be achieved to prevent a reversal of gains in this area.

In the case of RMNCH, the impact of consumable availability is far more evident, such that none of the expenditure scenarios considered would be able to stabilise the health burden in this area if not accompanied by significant invest in consumable access. This is due to generally low levels of consumable availability in this area of health [[2].](#_bookmark52)

Finally, in the case of NCDs, the effect of consumable availability is mostly negligible, suggesting again that reach and scope of currently implemented services may be a more significant barrier to an effective translation of expenditure into health outcomes (see discussion in the Results section).

It may be expected that, in reality, a transition between a present-level and perfect consumable availability would be gradual and a function of the yearly expenditure growth considered. We postpone these considerations to a future analysis.

**References**

1. Hallett TB, Mangal TD, Tamuri AU, Arinaminpathy N, Cambiano V, Chalkley M, et al. Estimates of resource use in the public-sector health- care system and the effect of strengthening health-care services in Malawi during 2015–19: a modelling study (Thanzi La Onse). The Lancet Global Health. 2024 Nov. Available from: <https://www.sciencedirect.com/science/article/pii/S2214109X24004133>.
2. Mohan S, Mangal TD, Colbourn T, Chalkley M, Chimwaza C, Collins JH, et al. Factors associated with medical consumable availability in level 1 facilities in Malawi: a secondary analysis of a facility census. The Lancet Global Health. 2024;12(6):e1027-37. Available from: <https://www.sciencedirect.com/science/article/pii/S2214109X24000950>
