# Supplementary material for: The potential impact of declining development assistance for health on population health in Malawi: A modelling study
Source: PLoS Med. 2025 Aug 21;22(8):e1004488. doi: 10.1371/journal.pmed.1004488 (PMC12370021; doi:10.1371/journal.pmed.1004488)
Supplement: S3 Text — (DOCX) [file pmed.1004488.s003.docx]

**IHME forecasts of health expenditure**

Two of the scenarios considered in our analysis, namely the “< GDP growth” and “<< GDP growth” scenarios, seek to approximate the lower and upper bounds in the 95% uncertainty interval in the forecast reported by the IHME, calculated by taking the 2.5th and 97.5th percentile of 1000 estimated random draws [3] (see Methods section [).](#_bookmark0) In this section, we discuss how we obtained these approximations.

The IHME provides projections on the fraction of total GDP allocated to health expenditures in Malawi by four financing source categories: government, DAH, out-of-pocket, and prepaid private, shown in Fig. [C.6.](#_bookmark18) In this work, we focus solely on the combined contribution of government and DAH expenditure, and therefore only consider those sources combined. Because our scenarios assume, for simplicity and interpretability, that *g*_fHE_ is constant over the period considered, we average the fractional change in the IHME projections in order to get an equivalent estimate of *g*_fHE_ at the end of the period, in 2040.

Included in the IHME forecasts is emergency COVID-19 funding for the years 2020–2021. The COVID-19 pandemic is however not included in the TLO model, therefore when calculating the average fractional change in *f*_HE_ from the IHME forecasts we extrapolate backwards past 2022 in order to “smooth-out” this emergency funding, as shown by the dashed lines in Fig [C.6.](#_bookmark18) The values obtained from these projections are *g*_fHE_ ∼ -1.4 and -3% for the upper and lower bound respectively, which are approximated by our “< GDP growth” and “< GDP growth” scenarios respectively, as shown in the same figure.


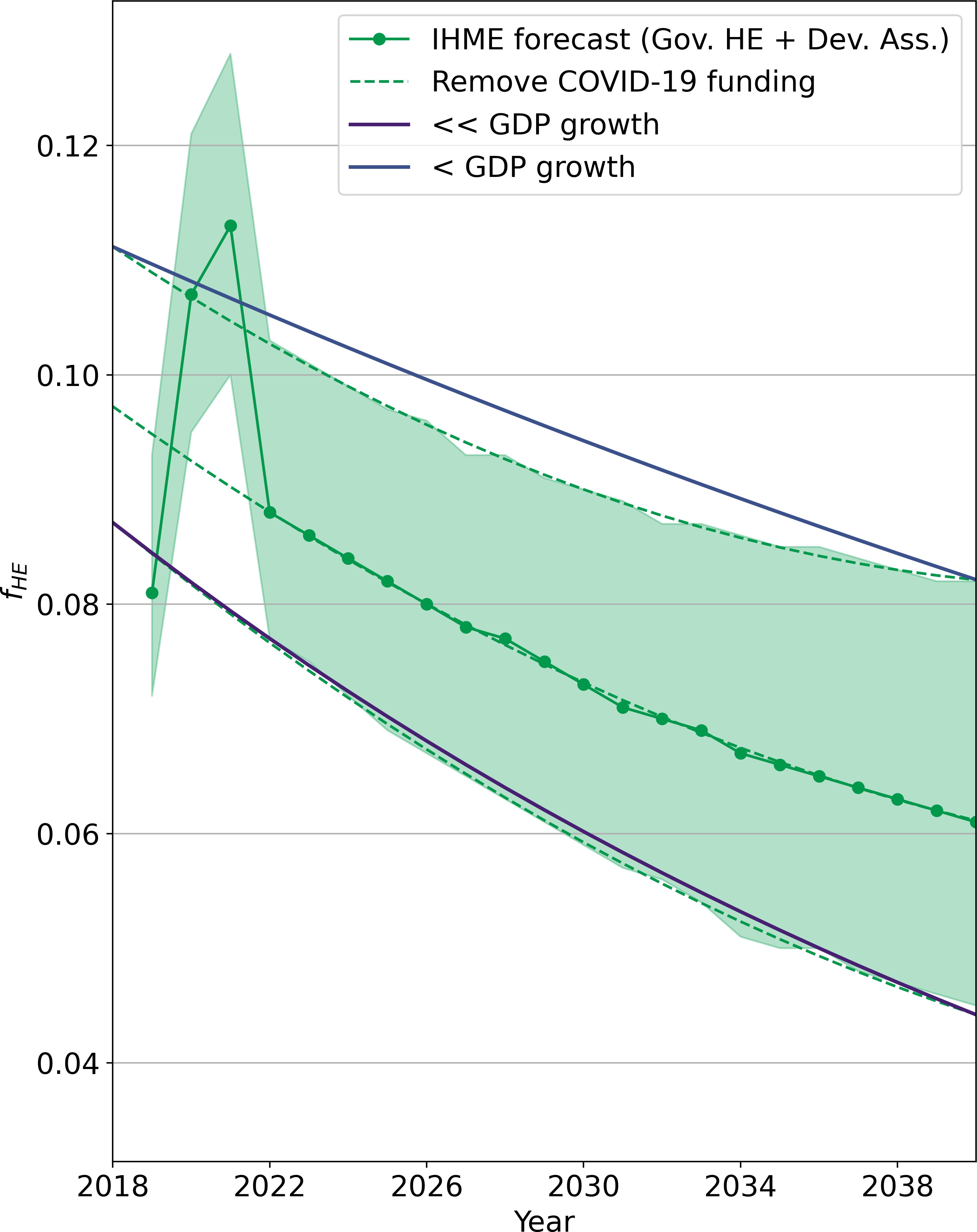


Fig C.6: Dashes lines show the best-fit to the mean, upper, and lower bounds of the projections for the combined government and DAH expenditures, extrapolated backward in time between 2018 and 2021 to “smooth out” COVID-19 emergency funding. The fractional change in the upper and lower interpolations are averaged over the entire period to produce the two scenarios that will approximate IHME forecasts in our analysis (“<< GDP growth” and “< GDP growth” respectively).
