# Supplementary material for: The potential impact of declining development assistance for health on population health in Malawi: A modelling study
Source: PLoS Med. 2025 Aug 21;22(8):e1004488. doi: 10.1371/journal.pmed.1004488 (PMC12370021; doi:10.1371/journal.pmed.1004488)
Supplement: S4 Text — (DOCX) [file pmed.1004488.s004.docx]

**Time evolution of individual causes of ill health**

In this section, we show the time evolution of the individual causes of ill health discussed in the Results section [3.2](#_bookmark7) under each of the funding scenarios considered. In Fig [D.7](#_bookmark8) we show HIV/AIDS, TB, and Malaria; in Fig [D.8,](#_bookmark19) we show all RMNCH causes; and in Fig [D.9,](#_bookmark26) we show a breakdown of all NCDs as well as the contribution of RTIs.


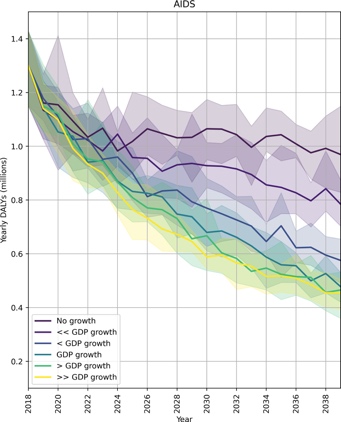

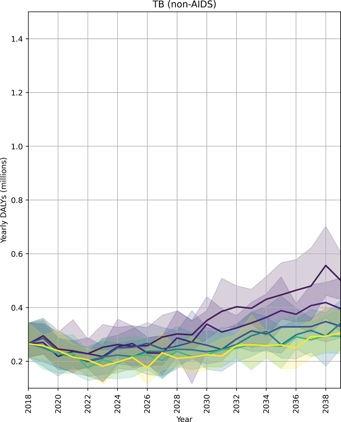

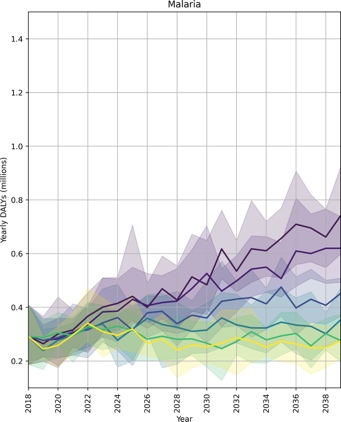


Fig D.7: Time evolution under each funding scenarios for HIV/AIDS, TB, and Malaria.


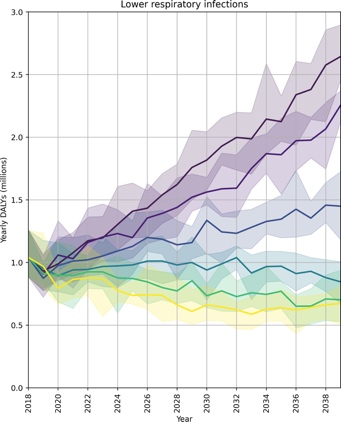

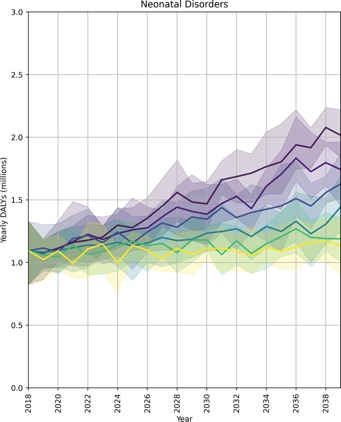

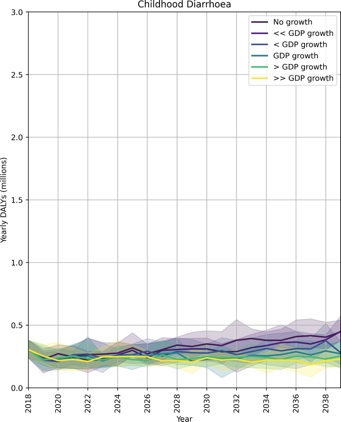


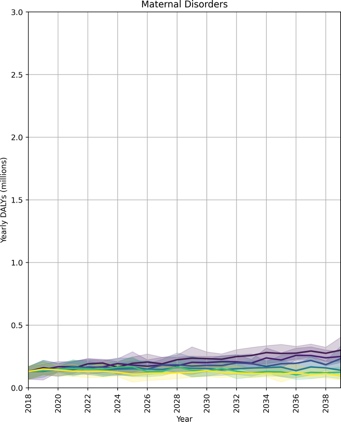

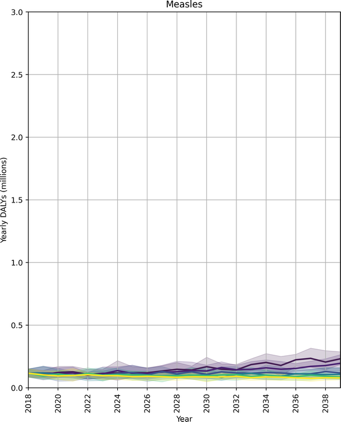


Fig D.8: Time evolution under each funding scenario for the RMNCH category.


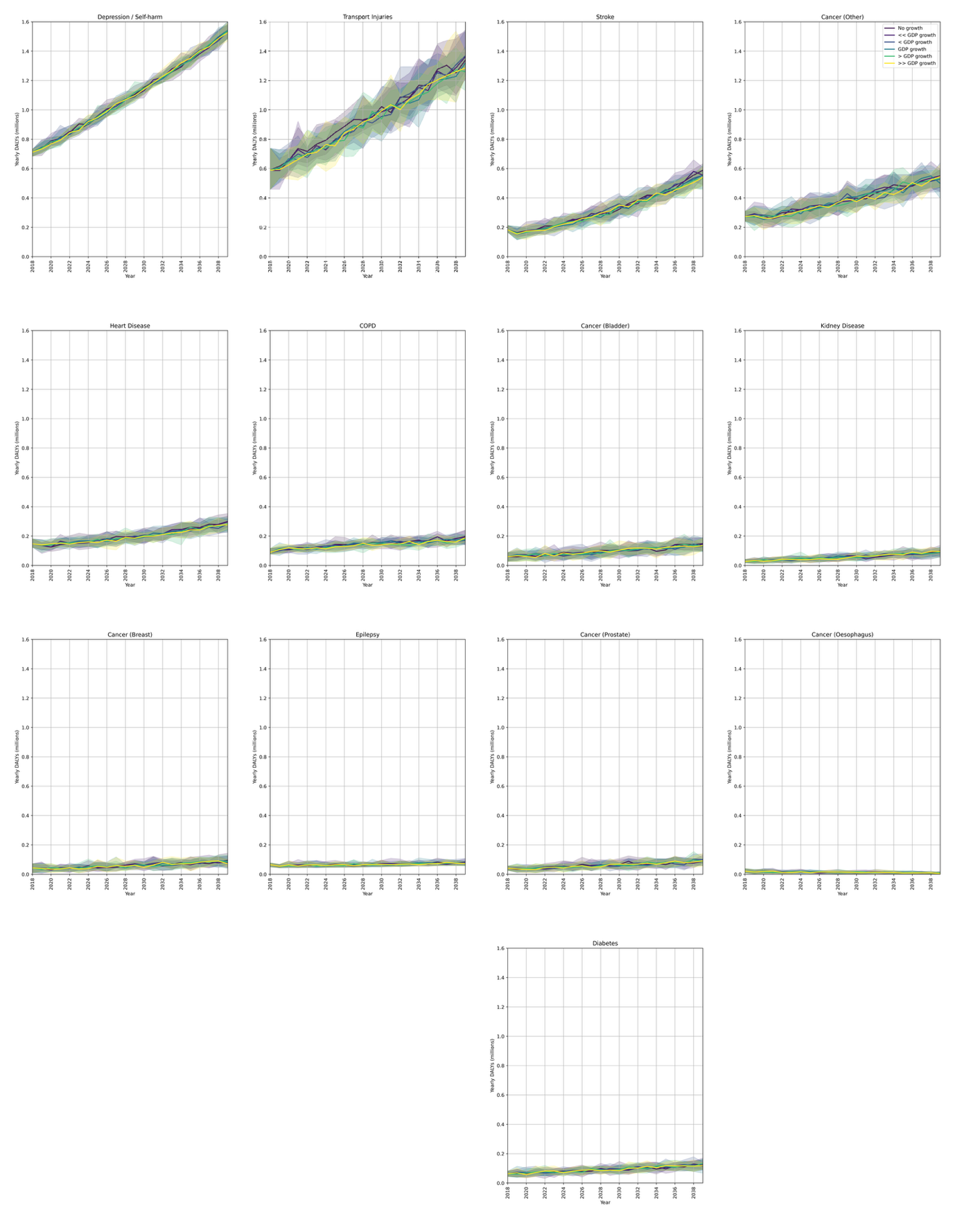


Fig D.9: Time evolution under each founding scenario for the NCDs and RTIs category.
