# Supplementary material for: The potential impact of declining development assistance for health on population health in Malawi: A modelling study
Source: PLoS Med. 2025 Aug 21;22(8):e1004488. doi: 10.1371/journal.pmed.1004488 (PMC12370021; doi:10.1371/journal.pmed.1004488)
Supplement: Attachment — Submitted filename: Response to Editor and Reviewers - PMEDICINE-D-24-03289R1.docx [file pmed.1004488.s007.docx]

We would like to thank the Editor and the Reviewers for taking the time to provide us with thorough and extensive feedback on the draft, which has undoubtedly allowed us to improve its quality, and which has given us interesting and important insights on the issues considered by our work. We provide detailed replies to each of the comments in more detail below, and have highlighted all changes in the resubmitted draft.

We believe the relevance of this work has increased considerably since the first submission, because of its contribution to showing - for the test case of Malawi - the risks to population health that come from declining in international funding. To address this, we note that we have now updated the abstract and discussion section to better guide the reader as to how our findings relate to recent changes in the international funding landscape.

We also highlight the following minor changes:

- The original section 4.1 (“Assume incidence of NCDs”) has now been moved to the end of the Discussion section, to give higher priority to other discussion points;
- We have rationalised the use of % vs actual factor value when defining the yearly expenditure growth parameter;

The research question is important, but there are limitations in the manuscript in its current form.

1. In its current form, there is limited generalisability outside of Malawi. Malawi also has certain unique population, healthcare, and economic features which mean that findings could only be generalised to other countries in the region or globally with extreme caution. Considerable editing and contextualisation in the introduction and discussion would help here.

We believe the strength of our approach lies in its very calibration to Malawi-specific factors, including all those mentioned by the Editor (population properties, healthcare, economic features), which ensures a tailored and contextually relevant analysis. We agree with the Editor that the results of this analysis should not be naively generalised beyond Malawi, and indeed we refrain from making any claims about the generalisation of our findings to other countries in the text, and only ever discuss our findings in the context of Malawi. Following the Editor’s recommendation, we have added the following considerations in the Discussion section:

*“The strength of this modelling approach indeed lies in its calibration to Malawi-specific factors, including population properties, healthcare structure, and epidemiological landscape, which ensures a country-tailored and contextually relevant analysis. The generalisability of these findings beyond Malawi, therefore, is unknown. However, by highlighting the potential consequences on health outcomes of reductions in DAH for Malawi, we hope to underscore the urgency of conducting similar analyses in other countries in the region and beyond that also rely heavily on foreign aid, and therefore are similarly vulnerable to its decline.”*

2. The authors should work to improve accessibility and clarity in their reporting, to ensure the broad audience of PLOS Medicine can understand what has been done, and why it is important.

We have taken this comment on board, and have tried to improve accessibility and clarity of the text in the revised draft. In particular:

- We have clarified the scope of our conclusions (see response to Editor comment #1)
- We have clearly outlined in the methodology all medical conditions explicitly included in the model, as well as the approach used to account for the health burden of conditions not directly modelled (see response to Editor comment #4);.
- We have clarified where readers can access detailed and comprehensive references of how individual medical conditions were modelled and calibrated (see response to Editor comment #4);
- We have added considerations on how modelling-based analyses can contribute to the wider deliberative process of policy design (see response to Editor comment #5);
- We have clarified assumptions around gGDP and gfHE values considered (see response to Reviewer #1, comment a)
- We have clarified why the impact of health expenditure on consumable availability is not currently considered in the analysis (see response to Reviewer #1, comment b);

If the Editor would like us to include any other clarifications in addition to these, we are willing to do so.

3. Reporting of methods and results are very brief currently, and would need to be substantially expanded to allow readers (and particularly general medical/public heath readers not in the field of development/development economics) to appraise the quality and rigour of the work that has been done.

We have taken this comment on board, and have expanded on the methods, discussions, and results in the reviewed draft as suggested. Please see response to previous comments for a detailed list of changes introduced. If the Editor would like us to include any other clarifications in addition to these, we are willing to do so.

4. In particular, some model inputs were not clear to me. For example, the impact on NCDs was mentioned, but it was not clear from this manuscript which NCDs contributed data. Trauma and road traffic injuries are a major contributor to ill health and healthcare utilisation in Malawi, particularly amongst the working age population. But I didn’t see whether they were counted as contributors. To be clear, this is only one example, and I could think of many others (eg air pollution as a “risk factor”, among others). The set of risk factors and conditions included may be comprehensive as the authors state, but I couldn’t find this information in the manuscript - currently there is only a link to another pre-print reference. The methods and sources of data for this manuscript should be described in full, and stand on their own.

We agree with the Editor that the transparency of the model inputs and clarity around where the reader can access further information should be improved. To do so, we have now added the following text to section 2.1.:

*“The medical conditions explicitly modelled in this analysis, in particular, include major infectious diseases such as human immunodeficiency virus (HIV)/acquired immune deficiency syndrome (AIDS) [7, 8], tuberculosis (TB)[7,8], and malaria [8]; non-communicable diseases (NCDs), such as cancers (including bladder, breast, oesophageal, prostate, and the combined effect of all others), cardio-metabolic disorders (including Diabetes Type 2, Hypertension, Stroke, Ischemic Heart Disease, Myocardial Infarction), chronic obstructive pulmonary disease (COPD), depression, epilepsy; road traffic injuries (RTIs) [9]; and the most prevalent conditions related to maternal and newborn health [10] as well as major causes of ill health among children under five years of age, including acute lower respiratory infections (ALRI) [11], measles, childhood diarrhoea, and stunting. A comprehensive, versioned documentation of all modelling assumptions for each of these conditions, including assumed risk factors and a review of all data sources and references adopted in each case, is available in the publicly-accessible repository of the model version used in this analysis, under docs/write-ups. This includes documentation (under the “Lifestyle” module) detailing how the model incorporates various social determinants of health, such as education level, wealth, marital status, and urban or rural residency. Finally, the incidence of all causes of ill health not directly modelled through an individual based approach, accounting for approximately ~19% of deaths and ~28% of disability-adjusted life-years (DALYs) documented in Malawi between 2015-2019, is captured using Global Burden of Disease (GBD) projections (see [6] for details). More details on the model can be found in [6] .”*

Furthermore, under section 3.1, we have added the following sentence: “*In Fig. 1 (left plot), we show the evolution of yearly DALYs incurred under different hypothetical scenarios of health expenditure, including all causes listed in section 2.1 and the correction from GBD projections from all remaining causes not explicitly included, as discussed in the same section.”*

While we understand that the Editor has suggested that the methods and data sources used should “stand on their own”, our motivations for relying on references and not replicating in this manuscript methods and data discussions available from other sources are:

1) To prevent duplication of previously published material and to clearly distinguish the novel methodology introduced in this paper from that already presented in prior publications.
2) To balance presenting clear and concise methods and findings in this manuscript while ensuring readers have access to comprehensive model details that cannot be fully summarised here (the docs/write-ups/Copd.docx reference, which explains the workings and calibration of one particular condition (COPD) only, is alone 15 pages long.)

Finally, we note that a detailed breakdown of the “NCDs” category considered in the analysis is listed in the main text (“In Fig. 3 we show how three important areas of health are affected by different expenditure strategies, namely [...] iii) non-communicable diseases (NCDs), including chronic obstructive pulmonary disease (COPD), cancers, depression/self-harm, diabetes, epilepsy, heart disease, kidney disease, and stroke.”, and in the caption to Fig. 3 (and NCDs, including COPD, cancers, depression/self-harm, diabetes, epilepsy, heart and kidney disease, and stroke”). Although Road Traffic Injuries (RTIs) had initially not been included in the NCDs category, they have now been added to the updated version of Fig. 3, at the request of the Editor.

5. The authors should provide greater critique of sources of input data. Particularly they should discuss whether GDB estimates (given the GBD’s input data) have sufficient validity and accuracy for Malawi to justify the level of precision of their estimates.

A comprehensive critique of sources of input data used in the calibration of the model is included in the model’s overview paper, Hallet et al 2024 (which we note is no longer in pre-print, but now published in Lancet Global Health). This reference is cited in this manuscript’s methods section (“All these factors are modelled explicitly and self-consistently, and are extensively calibrated to available data in the period 2015 – 2019 (see [6] for details).” ). In particular, processes regarding how the quality of data was assessed and how the model was calibrated using a variety of data sources are provided and discussed in the appendix (pp 100–263). We emphasise here that GBD estimates were not the only sources of data used for calibration: WPP, Malawi census, disease specific data sources like WHO vaccine coverage estimates, Malawi Population-based HIV Impact Assessment (MPHIA) survey, etc., as well as national records of service delivery from DHIS2, were all considered, and indeed nationally representative data was always preferred to GBD estimates in the calibration process, as clarified in the documentation provided (see reply to Editor comment #4).

In order to avoid replication of previously published peer-reviewed content, we have opted to cite rather than explicitly include these considerations in this manuscript.

6. The authors make a strong call in the discussion section for this modelling approach to be more widely used in development and healthcare policy. However, they do not reflect on the ethical issues around resource allocation based on these outputs. Allocation based on modelling like this may or may not be “fairer” than current approaches, but the authors should at least tackle this question in the Discussion. Specifically, from the results of their research, what would they recommend the international donor community and Malawi ministry of health fund differently over the next 10-20 years, and what would the likely consequences (positive and negative) of this be? What if this approach was applied in other countries in the region? Right now, (somewhat simplistically) conclusions reads as “we developed a very sophisticated way of predicting impact of funding, and countries should use it”.

We would like to clarify that our analysis in this manuscript does not aim to inform resource allocation within the healthcare system, but rather aims to predict health-system wide consequences of potential contractions or expansions in the overall level of funding for healthcare *assuming a present-day allocation* of available resources. We therefore cannot draw any conclusions as to what the donor community or the Malawi Ministry of Health should / should not fund differently in the next 10-20 years based on this analysis. As discussed in reply to the Editor’s comment #1, we also don’t make any claims as to the generalisability of these findings to other countries. We have however added the following considerations to the Discussion section:

*“For the first time, this modelling approach enables a health system-wide estimation of the potential impact of different hypothetical health system funding scenarios on population health, using a model extensively calibrated to the local context. Moreover, the Malawian Ministry of Health has been closely engaged from the earliest stages of the TLO model’s development and the conception of this analysis. As a result, this modelling approach provides valuable, context-sensitive scientific evidence [19] to support the wider and complex deliberative process of public healthcare policy design [20, 21].”*

-----------------------------------------------------------

Comments from the reviewers:

Reviewer #1: The Thanzi La Onse (TLO) model offers an innovative framework to simulate various health expenditure scenarios, yielding valuable insights into the projected health burden and resource needs within Malawi's healthcare system. However, despite the model's comprehensive approach, specific methodological aspects require enhanced transparency and rigor to ensure greater clarity and reliability in the findings. My comments are as in the following.

Major comments:

a)	Several assumptions are made regarding the expansion of human resources for health (HRH) based on GDP growth rates. These assumptions need further justification, particularly the assumption of constant GDP and fraction of GDP allocated to health (fHE) over the 2019-2040 period. Given the volatility in these figures, assumptions may need to be adapted or justified with additional context.

We would like to clarify that we do not assume a constant GDP or a constant fraction of GDP allocated to health (fHe) over the simulated period. Rather we assume:

- A constant rate of yearly GDP growth at 4.2%; and,
- A range of yearly percentage change to fHE, ranging from -3% a year (meaning that fHE is shrinking at a rate of 3% a year) to +3% a year (meaning that fHE is increasing at a rate of 3% a year).

Following the Reviewer’s comment, we have now significantly expanded section 4.1 to review our assumptions around fHE and GDP growth rates, which we report here:

*“The significant uncertainty surrounding future trends in GDP, DAH, and fHE, especially over extended periods of time, is inevitably reflected by any long-term projections assumed in this analysis.*

*Our analysis therefore deliberately considered a wide range of possible yearly expenditure growth scenarios, and remained agnostic as to which is more likely to be realised in the future, focusing instead on quantifying the population-health consequences in each case. For example, while the range of gfHE values considered included IHME projections, this was deliberately extended to capture a wider range of potential annual changes in fHE.*

*We further emphasise that in this analysis, it is the assumed annual expenditure growth — which reflects the combined effects of gGDP and gfHE - which determines the HCW capacity expansion. This allows for flexibility in interpreting each scenario under different gGDP and gfHE assumptions, by adjusting one parameter while proportionally rescaling the other. For example, the “< GDP growth” scenario (gGDP = 4.2%, gfHE = -1.5%) could equally represent a scenario where gGDP = 2% and gfHE = 0.62%. And while in designing these scenarios we assumed that these parameters could vary independently of one another, each scenario could therefore easily be reinterpreted as reflecting any level of correlation between them, which may better reflect how these two parameters evolve in reality.*

*Finally, we assumed that gGDP and gfHE remain constant throughout the simulated period for each scenario. In doing so, we have chosen to prioritise interpretability over complexity: accounting for fluctuations in gGDP and gfHE over the simulated period would have introduced complex temporal dynamics in the disease burden, influenced by the timing, magnitude, and cumulative effect of prior investment, as well as the intrinsic transmission dynamics of the simulated infectious diseases. Such interactions would have significantly complicated result interpretation and scenario comparison, particularly given the arbitrary nature of the timing and magnitude of any simulated fluctuations. Given that this is the first study of its kind to undertake this type of analysis, we therefore believe this to be a reasonable assumption at present, and postpone a more detailed analysis of the effect of these fluctuations to a later study.”*

Finally, the Reviewer correctly points out that a number of assumptions were made regarding how HRH expansion tracks the level of investment into health. We note that these are extensively reviewed in the Discussion section 4.2.

b)	The manuscript notes an assumption of perfect consumable availability throughout the model's projections. Although a sensitivity analysis is presented for this assumption, it would be beneficial to integrate more nuanced scenarios (e.g., variable consumable availability in response to funding changes) to improve the model's realism and applicability.

We agree that a more nuanced analysis of consumable availability under varying funding scenarios would be highly valuable. The reason we did not include this at this stage is due to the complexity of accurately modelling the relationship between financial investment and improvements in consumable availability. The costs associated with strengthening supply chains, ensuring effective stock management, and minimising shortages remain at present poorly understood. Nevertheless, our sensitivity analysis demonstrates that our key conclusions hold across a wide range of assumptions regarding consumable availability, including both extremes of consumable availability (observed present-levels sustained into the future, or perfect consumable availability). We have now included the following statement in the discussion:

*“This analysis assumed perfect consumable availability in all disease areas throughout the entire simulated period. A sensitivity analysis (see Appendix B) was conducted on this assumption, testing a scenario in which consumable availability instead remains fixed at its present-day level [6] without any improvement.*

*For mostly vertically funded programmes such as HIV/AIDS, current levels of relevant consumable availability in Malawi are already extremely high [7], and generally higher than those in other disease areas [6]. The resilience of the health burden due to this cause captured by our analysis is therefore contingent on a high probability of relevant consumables being available. Any shocks or disruptions to relevant supply chains could therefore undermine this finding, as discussed in more detail in section 4.4.*

*Finally, we note that a more nuanced analysis of consumable availability under varying funding scenarios would be highly valuable. Due to the complexity of accurately modelling the relationship between financial investment and improvements in consumable availability, we have however refrained from doing so at this stage: indeed, the costs associated with strengthening supply chains, ensuring effective stock management, and minimising shortages, remain at present poorly understood.”*

c)	The TLO model is complex, capturing various factors affecting health in Malawi. Yet, there is limited discussion on the specific calibration of model parameters to real-world data (2015-2019). Including a summary of how well the model's outputs match historical data would strengthen its validity and improve transparency.

As mentioned in our response to point 4 raised by the Editor, we have now clarified in the manuscript where readers can find a detailed description of the assumptions underlying the modelling of each medical condition included in this analysis, along with the data sources and references used in each case (including a published paper with an extensive technical appendix, our public code repository, and a website where data sources and parameters can easily be browsed). Because of the high level of detail captured in the model, we have opted not to replicate information that is already available in other peer reviewed publications on this model, and instead only discuss methods that are innovative to this specific analysis. The calibration of the overall model to historical data, in particular, is extensively discussed in the model’s overview paper (Hallett et al 2024), which is cited in the text when the calibration of the model is mentioned “(All these factors are modelled explicitly and self-consistently, and are extensively calibrated to available data in the period 2015 – 2019 (*see [6] for details*).”

Please refer to our response to comment 4 raised by the Editor for more details.

d)	The model enforces HRH constraints by limiting healthcare delivery based on available patient-facing time, yet does not fully account for productivity variations over time.

This is a limitation of our analysis that we acknowledge in our discussion section (“we therefore did not account for i) possible variations in HCW productivity due to the expansion of existing cadres or other influencing factors”). While we agree that accounting for variations in productivity would be an extremely important addition to this analysis, more empirical analysis is required to understand how productivity of healthcare workers varies as a function of environmental factors (such as overall demand for care and number of healthcare workers available), policy decisions, adoption of incentives, and improvements in available technologies, before this can be incorporated into a modelling approach. Such empirical studies are currently under way and we hope to include them in our modelling framework once we are confident we have a sufficient empirical understanding of this dynamic.

We have added this point to the discussion, as follows*: “Accounting for variations in healthcare worker productivity would also be an extremely important addition to this analysis; while this is a focus of future work on the model, we believe more empirical analysis is first required to better understand how productivity of healthcare workers varies as a function of environmental factors (such as overall demand for care and number of healthcare workers available), policy decisions, incentives, quality of management, and improvements in available technologies..”*

e)	The model appears to assume uniform availability of HRH across all districts and facility levels, which may not be reflective of reality. More granular modelling, with variations in HRH distribution and healthcare access across different regions, would increase accuracy.

While we do not make this assumption, we agree with the Reviewer that this was not made sufficiently clear in the description of the methods. We have clarified this point in section 2.2 as follows:

*“Initial HRH capabilities assumed are extensively calibrated to available data, disaggregated by medical cadre, facility level, and district (see She et al 2024 for details). In each scenario, we then assume that capabilities in each category are expanded by the same proportional factor, such that the initial, relative distribution of HCWs is maintained throughout the expansion period.”*

The limitations of the latter assumption are already acknowledged in the discussions sections as follows: “The [added: initial relative] distribution of HCWs across different cadres, facility levels, and districts remains constant as HRH capabilities are expanded. This strategy for resource allocation may be far from optimal. A more targeted expansion of capabilities in particular districts/facility levels/types of cadres may indeed result in a higher return in health from the same expenditure. This analysis should therefore be seen as a “lower bound” to the health benefit that could be obtained from the same expenditure.”

f)	While the manuscript reports 95% confidence intervals for projections (e.g., DALYs), there is limited detail on how these intervals were computed. Clarification on the statistical methods used to calculate the intervals, including any resampling or bootstrapping techniques, would strengthen the reliability of these estimates.

We thank the Reviewer for pointing that this was not sufficiently made clear in the text. We have now added the following clarification to the method section:

“The initial representative population size assumed in 2010 is of 100,000 individuals, while results reported are scaled to the true population size in Malawi in 2010 of 14.5 million individuals. Each scenario was simulated 10 independent times, each with independent random draws to capture stochastic uncertainty; this specification was found to be sufficient to give stable estimates of the mean and variance of the health burden obtained under each cause included in the simulation over independent realisations. *For each of the scenarios, we plot the mean and the 95% interval across the 10 model realisation. The latter is obtained by calculating the 2.5% and 97.5% quantiles in the recovered outcomes assuming a linear interpolation in the distribution.”*

g)	Although the model has undergone internal validation, it lacks external validation against other health projection models or empirical data. Benchmarking projections against independent data sources or similar models could add credibility and confidence in the findings.

While we share the Reviewer’s interest in a comparative exercise, we are not aware of any other analyses, empirical or otherwise, that have estimated the health burden that should be expected in Malawi - or any other country - due to variations in funding streams to the healthcare system. We are therefore unable to carry out this requested external validation at this time. We have however explicitly noted in the revised manuscript.

*“At present, no other study has directly linked future healthcare spending implications to population health outcomes in Malawi or other countries. We are therefore unable to conduct a comparative exercise on our results.”*

h)	The manuscript presents different growth scenarios for health expenditure, but additional statistical comparisons between scenarios would be valuable. For instance, a quantitative comparison (e.g., through effect size calculations or relative risk reduction) of health outcomes across scenarios could help quantify the impacts of varying health expenditure levels more precisely.

In Fig. 1b (right panel) we provide a quantitative comparison of the evolution of life expectancy over the simulated period between scenarios, which provides a summary statistic of risk reduction at the individual level from all causes simulated, in each of the scenarios considered, as requested by the Reviewer. At a population level, the relative risk reduction in the form of % DALYs averted is included in Fig 2, right panel. We would however be happy to include additional summary statistics at the request of the Reviewer.

i)	Although the model is open-source, detailed documentation on data inputs and assumptions in the supplemental materials would support reproducibility. Clear data availability statements, particularly for health outcome and HRH projections, could enhance transparency.

Following comment 4) of the Editor, we have now provided additional information in the manuscript on where readers can access detailed documentation on data inputs used in the model, both for simulated medical conditions (see reply to Editor’s comment 4) and on HRH (see response to comment e) by Reviewer #1). All of this information can be directly and comprehensively accessed from the publicly available repository of the model provided. Model outcomes can be reproduced from the publicly available repository. We have added tags to the repository and guidance for users to make this as easy as possible. If the Reviewer and the Editor would find this useful, we would be happy to create an additional public repository including the postprocessed simulation outcomes.

Additional comments:

j)	The model assumes consistent effectiveness of interventions throughout the projection period. Yet, changes in intervention efficacy (e.g., due to drug resistance, policy shifts) can impact outcomes. Incorporating variable effectiveness rates or sensitivity analyses on intervention efficacy would enhance the model's adaptability to real-world conditions.

The major innovation in this work is to show how health financing might lead to changes in population health projections at a “macroscopic” level. This has never been done before and we wish to make that analysis and its presentation as clear as possible.

Therefore, given

i) the wide range of interventions included in the model, relating to 30 different medical conditions,

ii) the high level of uncertainty around policy shifts and innovation in treatment and prevention that could be expected over the period considered, and an absence of systematic evidence on these trends,

iii) the opposing effects of some of the sources of this uncertainty (e.g. drug resistance decreasing while innovation enhancing the impact of interventions),

We believe this kind of analysis to be prohibitive at this stage, and better suited to a single disease analysis.

We have however added a statement to clarify that our analysis is conducted under assumptions of present-day level of effectiveness of interventions, as follows.

*“Finally, we note that the assumed efficacy of specific interventions is informed by data and inferred through the calibration process. The effectiveness of interventions in reality can, of course, evolve over time, influenced by factors such as the emergence of drug resistance to some infectious pathogens, advancements in treatment, or changes in policy. In this analysis however we do not capture the uncertainty around the future effectiveness of the treatments considered, and instead assume this to be constant with time.”*

k)	While the model assesses the DALYs for general categories (e.g., HIV/AIDS, NCDs), the aggregation of different health conditions within these broad categories limits the granularity of the findings. Further breakdown of health outcomes could allow for more targeted conclusions, particularly given the differing health service requirements across diseases like diabetes versus COPD within the NCDs category.

We have added, in Appendix D, a cause-by-cause breakdown of the time evolution of yearly DALYs under different expenditure scenarios. We hope this strikes the right balance between focusing the discussion on population-wide outcomes while providing the reader with access to detailed information on how individual diseases contribute to this burden.

l)	While consumable availability is addressed as either perfect or present-day, this binary approach may not capture the complexity of real-world supply chain challenges. A more nuanced approach, considering gradual improvements or degradation in consumable supply with changing funding levels, would provide a more granular view of system constraints.

Please see reply b) to Reviewer #1.

m)	While the TLO model captures demographic growth and disease incidence, it does not appear to fully account for social determinants of health or regional disparities within Malawi. Including these variables or discussing their potential impact on projections would improve the model's applicability to varied demographic settings within the country.

The model accounts for a number of social determinants of health, including level of education, level of wealth, marital status, and urban/rural status, as reviewed in the “Lifestyle” module reference of the model’s documentation (we have now clarified this in the text, see response to Editor’s comment #4). Regional disparities within Malawi are accounted for at a district level both in terms of healthcare resources available (both in the form of human resources for health and consumables) and incidence of disease.

n)	The manuscript would benefit from discussing projection accuracy metrics, such as mean absolute error (MAE) or root mean square error (RMSE), based on past health outcomes.

We wish to clarify that this analysis is not a predictive exercise whose accuracy can be evaluated against future data. Instead, it translates various hypothetical funding scenarios into their potential impacts on population health. This approach, which has not been undertaken before, represents the core innovation of our work.

The process of calibration of the Thanzi La Onse model used in this analysis itself is extensively discussed in its peer-reviewed overview paper (Hallett et al 2024). In particular, processes regarding how the quality of data was assessed and how the model was calibrated are provided in the appendix (pp 100–263). Please see reply to Editor’s comment #5 for more details.

o)	The manuscript could enhance its scenario comparison by quantifying the differences between them with confidence intervals. This would help in clearly illustrating the statistical significance (or lack thereof) between the health outcomes across different expenditure scenarios.

Please find a quantification of the difference between scenarios and respective confidence intervals in Fig. 2b.

p)	A more detailed description of the data sources used to populate the model would improve transparency. This should include the quality, limitations, and temporal range of each dataset, particularly for health burden and expenditure data.

Datasets considered for model calibration are extensively discussed in the model’s overview paper, which is cited in the main text when the model is first introduced (Hallet et al 2024), and in the documentation provided on each of the simulated medical conditions (see reply to Editor’s comment #4). We have also included additional references on the datasets used to inform the assumptions around healthcare worker availability in each district and at each facility level (see reply to Reviewer #1 comment e).

Reviewer #2: This article reports the results from a simulation modeling exercise focused on the potential impact of decreases in health spending on the health burden in Malawi. The authors are to be commended for applying their skill set and methodology on a relevant and important case study. The results from the study provide useful insights for stakeholders in the health sector in Malawi on the implications of various scenarios of health spending that could potentially materialize. It therefore offers policymakers and decision makers in the sector possible future outcomes that can be targeted with current actions. I provide some additional comments below to further strengthen the current manuscript.

Feedback

- Recommend adding text in the introduction or discussion to clearly highlight that the results highlighted in the study are based on hypothetical scenarios which may or may not occur depending on actions that are taken. Thus, emphasizing the importance of evidence-based decision making for readers and stakeholders in the sector.

In the introduction we clarified that ‘we consider hypothetical long-term health expenditure scenarios, *which may or may not realise depending on actions taken by both international and national funders and stakeholders*.”

- While the simulation platform's original documentation is referenced, I recommend a summary paragraph on the model components to orient readers who may be encountering this model for the first time.

This has now been added, please see the response to Editor’s comment #4 for more details.

- It may be useful for the readers to add text for why the IHME dataset rather than other alternative sources of this data is utilized in the study and also why the particular dataset is utilized and not the updated versions of the IHME dataset.

Projecting the future availability of health aid, especially over extended periods of time, is fraught with difficulty. The past month especially has clearly shown this. IHME is a well recognised and well cited source of evidence on global health projections, so we based the analysis on this whilst recognising the inherent uncertainty. As noted in our responses above, however, we have added the following note of caution in the discussion:

“*The significant uncertainty surrounding future trends in GDP, DAH, and fHE growth, especially over extended periods of time, is inevitably reflected by any long-term projections assumed in this analysis.*

*Our analysis therefore deliberately considered a wide range of possible yearly expenditure growth scenarios, and remained agnostic as to which is more likely to be realised in the future, focusing instead on quantifying the population-health consequences in each case. For example, while the range of gfHE values considered included IHME projections, this was deliberately extended to capture a wider range of potential annual changes in fHE*.”

Since the analysis was undertaken, updated IHME estimates became available. We have however verified that the updated estimates don't change the conclusion of our analysis, as illustrated below. We also stress that our analysis is not dependent *per se* on the IHME forecasts: while we have chosen some scenarios to assume values of gfHE that match those predicted by IHME 2021, the range of possible gfHE that we consider is wider and includes those forecasted by IHME 2024, as illustrated below. Given the high level of uncertainty in any such projections, especially at the moment (see for example the rapid and unexpected reduction in funding received through USAID), we believe this to be the most appropriate approach.

- Recommend adding a paragraph to the discussion that discusses the results from this exercise in context with either any relevant studies that discuss the implication of future spending and health outcomes in Malawi or perhaps in comparable neighboring countries.

Please see reply to Reviewer #1, comment g.

- You do this a bit in the section where you highlight the assumptions embedded in the choices available in the simulation model set up but I recommend also adding a paragraph to the discussion in which you discuss the other factors beyond human resources that impact disease burden covering factors in the health sector such as the availability of medicines etc. but also factors outside the health sector such as sanitation, employment etc. that impact health outcomes.

We note here that sanitation is included as a risk factor for some conditions and changes with time, using DHS estimates. Following the Reviewer’s comment, we have however added the following sentence to the discussion to acknowledge this limitation: “Finally, our analysis doesn’t capture factors beyond health system provision which may impact the country’s health outcomes and may reasonably be assumed to evolve during the simulated period, such as access to improved (or declining) infrastructure, education, and employment.”

Reviewer #3: This manuscript presents an interesting approach with innovative modeling and data analysis. To enhance its clarity and comprehensiveness, it would be beneficial to expand on the following areas:

* Methods:

o Provide more details on the scenarios presented in Table 1, specifically explaining how key parameters (e.g., gGDP, gfHE) interact. Are these variables simultaneously determined at certain levels?

Following the ReviewWe have significantly expanded this discussion in section Please see response to Reviewer #1, comment a).

o Does the model assume a constant productivity level for the health workforce, considering the projected increase in the number of health workers.

Please see response to Reviewer #1, point d).

*Results:

o Further elaboration on the modeling results related to HRH capacity is recommended, as this is a critical component of the theory of change presented. Specify the number of health workers required under each scenario and assess whether these projections are realistic given the country's existing capacity to train and deploy health workers, as well as current health labor market entry patterns. Consider discussing potential constraints and their implications for future health expenditures.

We acknowledge the limitation as to whether these projections of HRH expansions are realistic in the discussion section, as quoted here:

“In addition, we assumed that any expenditure – based on the above-listed assumptions – directly translated into an expansion of available patient-facing time. In this simplified approach, we therefore did not account for [...] ii) the fact that the realisation of such scenarios would require adequate and timely expenditures in training and recruitment of additional HCWs, as well as potentially the expansion of existing infrastructures, facilities, and equipment to accommodate the intake of new personnel. These costs may amount to a higher share of the healthcare budget currently allocated to these areas; fully capturing them while imposing a cap on overall expenditure may therefore limit the amount of HRH capabilities expansion achievable under the same assumed GDP and f_HE_ growth, as would potential wage increases over the simulated 22-year period. Once such additional costs are captured, such considerations could however easily be embedded in this analysis by refactoring the assumed growth/total expense incurred under each scenario considered.”

Finally, we note that in the text we reference the data source used to initialise the health worker force at the start of the simulated period in 2018. Given the very simple expansion of this initial workforce in the scenarios considered (by a fixed percentage every year across the simulated period) we believe that providing data on a year-by-year evolution of the workforce may be unnecessary.

* Implications:

o Outline specific policy recommendations derived from the analysis and results.

We have added the following to the conclusions:

“In particular, we find that current levels of expenditure on HRH as a fraction of GDP should be sustained in order to prevent a reversal of gains in many important areas of health, such as RMNCH, TB, and malaria. Furthermore, we find that the rising contribution of NCDs to the national health burden is currently largely unaffected by investments in HRH, suggesting that an expansion to the scope and reach of preventative and treatment programmes relevant to these diseases should be considered, if the rising contribution of these conditions is to be contained.”
