# Supplementary material for: The potential impact of declining development assistance for health on population health in Malawi: A modelling study
Source: PLoS Med. 2025 Aug 21;22(8):e1004488. doi: 10.1371/journal.pmed.1004488 (PMC12370021; doi:10.1371/journal.pmed.1004488)
Supplement: Attachment — Submitted filename: PMEDICINE-D-24-03289R2 Second Round Reviewers Response.docx [file pmed.1004488.s008.docx]

Reviewer #1:

a)	The manuscript mentions confidence intervals but does not explicitly state whether parametric or non-parametric methods were used to generate them.

We have now explicitly stated this as follows: *The 95% interval [Added:] is a non-parametric confidence interval, obtained by calculating the 2.5% and 97.5% empirical quantiles of the simulation outcomes using linear interpolation.*

b)	The study conducted sensitivity analyses on consumable availability, but incorporating additional scenario testing (e.g., simulating dynamic GDP growth rates over time) would enhance the study's robustness.

The motivation for not including scenarios with dynamic GDP/fHE growths is provided in the discussion section, as follows: *“Finally, we assumed that gGDP and gfHE remain constant throughout the simulated period for each scenario. In doing so, we have chosen to prioritise interpretability over complexity: accounting for fluctuations in gGDP and gfHE over the simulated period would have introduced complex temporal dynamics in the disease burden, influenced by the timing, magnitude, and cumulative effect of prior investment, as well as the intrinsic transmission dynamics of the simulated infectious diseases. Such interactions would have significantly complicated result interpretation and scenario comparison, particularly given the arbitrary nature of the timing and magnitude of any simulated fluctuations. We therefore believe this to be a reasonable assumption at present, and postpone a more detailed analysis of the effect of these fluctuations to a later study.”*

c)	Assessing extreme-case scenarios—such as economic downturns or donor funding reductions beyond IHME projections—could provide deeper insights into the resilience of the model's findings.

Our motivation for limiting the range of scenarios considered in this analysis is provided in our answer to point b). We note that we are currently in the process of expanding this analysis to specifically address the recent, sudden reductions in DAH resulting from PEPFAR/USAID defunding, specifically the potential decline in HIV/AIDS-specific services, both from a human resources for health and a consumables perspective. We believe this analysis is best suited for a dedicated publication to maintain the focus and scope of the current manuscript.

d)	While the model is calibrated to historical data, it does not explicitly assess predictive validity. The authors could strengthen their analysis by employing a back-testing approach, comparing past projections with actual outcomes (if available). Additionally, external validation using alternative datasets (e.g., WHO health expenditure and outcome trends in comparable countries) could improve the credibility of their projections.

We agree with the reviewer that this is currently a limitation in the analysis. A large data collection effort was undertaken in 2024 in Malawi specifically to produce datasets against which the TLO model could be validated. Data is currently being cleaned and analysed.

e)	The model does not appear to account for the broader economic impacts of health funding changes, such as effects on labour productivity, poverty reduction, or private-sector investment in healthcare.

The aim of this study was to quantify the impact of varying levels of public healthcare expenditure on population health. While we recognise that changes in health outcomes can, in turn, influence broader economic conditions, quantifying these economic impacts lies beyond the scope of this analysis. However, we acknowledge that such effects are likely to occur and could, in turn, influence population health outcomes. We acknowledge this in the following sections of the discussion:

Poverty reduction: *“Furthermore, while HRH constitutes one of the most important constraints to healthcare delivery and universal healthcare access [22, 23] we do not capture [...] how factors beyond health system provision which may impact the country’s health outcomes, such as access to improved (or declining) infrastructure, education, and employment, would evolve with time, [Added:], or themselves be influenced by public healthcare spending.”*

Private-section contribution: *“Finally, this analysis does not capture the contribution of prepaid private and out-of-pocket health expenditures to the overall health outcome of the population. The IHME forecasts that the relative contribution of these types of health expenditures to the overall health expenditure in the country will grow over this period, from an estimated 17% in 2019. [Added:] However, the future trajectory of prepaid private and out-of-pocket health spending is itself likely to be influenced by the level of investment in public healthcare, as changes in public provision may shape demand for private alternatives. ”*

Productivity:  *“We therefore did not account for i) possible variations in HCW productivity due to the expansion of existing cadres or other influencing factors,”*

and

*“Accounting for variations in healthcare worker productivity would also be an extremely important addition to this analysis; while this is a focus of future work on the model, more empirical analysis is first required to better understand how productivity of healthcare workers varies as a function of environmental factors (such as overall demand for care and number of healthcare workers available), policy decisions, incentives, quality of management, and improvements in available technologies.”*

f)	The authors assume constant health expenditure growth rates and fixed intervention effectiveness over time. However, real-world factors—such as medical inflation, technology adoption, and policy shifts—could significantly influence outcomes.

The assumption of constant health expenditure growth is motivated in response to Reviewer’s comment b).
With regards to the assumption of fixed intervention effectiveness over time, this is addressed in the manuscript as follows: “*The effectiveness of interventions in reality can, of course, evolve over time, influenced by factors such as the emergence of drug resistance to some infectious pathogens, advancements in treatment, or changes in policy. In this analysis however we do not capture the uncertainty around the future effectiveness of the treatments considered, and instead assume this to be constant with time.”*

g)	While the study estimates the health burden under different expenditure scenarios, incorporating an explicit cost-effectiveness analysis (e.g., cost per DALY averted) would enhance the policy relevance of the findings.

We agree that cost-effectiveness metrics such as cost per DALY averted could provide some additional policy insights. Our motivations for refraining from including it at this stage are as follows:

- The primary aim of this study was to assess the implications of externally determined changes in overall health expenditure (such as a decline in DAH) rather than to evaluate a broad set of programmatic options for resource allocation, for which cost-effectiveness analysis is most appropriate.
- In our analysis, we defined a “normalised health expenditure” for each scenario. Although this does not represent the actual financial costs required to implement each funding scenarios, it enables readers to assess the relative differences in expenditure across scenarios and compare them with the corresponding changes in health burden (see Fig. 2, top x-axes). While not a conventional cost-effectiveness analysis, this approach facilitates direct comparison of the health impact of incremental funding changes, offering insight into the marginal returns on investment in each case. Given that implementation efforts (such as training, recruitment, etc) and associated costs are not presently captured (as acknowledged in the manuscript), we believe this simplified approach to be more appropriate at this stage.
